# Along the Right Lines

**Published:** 1914-07

**Authors:** 


### Along the Right Lines.

"The New York City Health Department has extended the diagnostic station work of its venereal disease division by adding a Brooklyn station, and has continued its research in institutions. More than 13,000 laboratory tests for syphilis alone have been made during the past year. Many patients came to the department for diagnosis and advice, and if infected are sent to a physician for treatment, but the majority are sent for diagnosis by physicians who need laboratory assistance in making a diagnosis. The influence and value of such practical work cannot be overestimated. Other cities and a few States are planning to have or have already begun like work."—*Bulletin.*

The result of the campaign against social vice has been to demonstrate most emphatically the urgent necessity of just such medical supervision. If we are to pass laws requiring a medical certificate before issuance of marriage license, it will become obligatory on the part of the State to provide every facility for just such work. Considered from an economical standpoint alone, State diagnostic laboratories would prove a most profitable investment.

